# Real-time electrocatalytic sensing of cellular respiration

**DOI:** 10.1016/j.bios.2014.01.059

**Published:** 2014-07-15

**Authors:** Nga-Chi Yip, Frankie J Rawson, Chi Wai Tsang, Paula M Mendes

**Affiliations:** a School of Chemical Engineering, University of Birmingham, Edgbaston, Birmingham B15 2TT, United Kingdom; b Laboratory of Biophysics and Surface Analysis, School of Pharmacy, University of Nottingham, University Park, Nottingham NG7 2RD, United Kingdom; c School of Chemistry, University of Birmingham, Edgbaston, Birmingham B15 2TT, United Kingdom

**Keywords:** Biochemical oxygen demand assay, Ferrocene carboxylic acid, Electrocatalyst, Toxicity, Electrochemical assay

## Abstract

In the present work we develop a real-time electrochemical mediator assay to enable the assessment of cell numbers and chemical toxicity. This allowed us to monitor metabolism down to a single cell in a low cost easy to use rapid assay which is not possible with current technology. The developed assay was based on the determination of oxygen. This was made possible via the use of electrochemical mediator ferrocene carboxylic acid (FcA). The FcA showed distinctive catalytic properties in interacting with reactive oxygen species generated from oxygen when compared to ferrocene methanol (FcMeOH). A deeper insight into the chemistry controlling this behaviour is provided. The behaviour is then taken advantage of to develop a cellular aerobic respiration assay. We describe the properties of the FcA system to detect, in real-time, the oxygen consumption of *Escherichia coli* DH5-α (*E. coli*). We demonstrated that the FcA-based oxygen assay is highly sensitive, and using a population of cells, oxygen consumption rates could be calculated down to a single cell level. More importantly, the results can be accomplished in minutes, considerably outperforming current commercially available biooxygen demand assays. The developed assay is expected to have a significant impact in diverse fields and industries, ranging from environmental toxicology through to pharmaceutical and agrochemical industries.

## Highlights

• The electrocatalytic behaviour of ferrocene carboxylic acid (FcA) to quantitate oxygen is elucidated.
• FcA was used as a reporter to monitor oxygen use and production by bacteria and algae.
• FcA was used to monitor the metabolic rate of *E. coli* leading to the development of a rapid toxicity assay.

## 1 Introduction

Oxygen is essential for aerobic life, acting as the final electron acceptor in the respiratory pathway. Therefore, the rate of oxygen consumption is proportionally linked to cell numbers, viability, growth rate and the phase of cell cycle that an individual cell is in Fisher and Armstrong (1946) and Martin (1932). In the past decade, biochemical oxygen demand assays (BOD) were developed, specifically for the food and waste water processing industry, as a mean to determine and quantitate the presence of aerobic microorganisms in food and water sources. The current BOD methodologies can be sub-divided into two main approaches, fluorescence-based and electrochemical.

Fluorescence-based BODs have been based upon using dual-dye systems, whereby, two fluorophores are used simultaneously (Wang et al., 2012). A dual-dye system was introduced into biological studies to remove inconsistencies produced by dye loading, leakage, bleaching and cell movement artefacts (Ashby and Tepikin, 2002; Sekar and Periasamy, 2003; Wang et al., 2012; Bradshaw and Knaide, 2010). There are several major disadvantages with using fluorophores in BOD systems. Fluorophores are commonly cytotoxic themselves (Alford et al., 2009). Additionally, fluorophores are extremely sensitive to light and are prone to photobleaching leading to the loss of active fluorophores over time. Fluorescence-based BODs are only ratiometrically quantitative and not absolutely quantitative (Cabrerizo et al., 2010; O׳Neal et al., 2004; Wang et al., 2012). In addition, ratiometric quantification is not very sensitive and can only be useful for comparing general experimental variables, such as the drop in relative signal intensity. Moreover, results obtained via ratiometric quantification tend to have large errors associated with collected data due to many inter and intra experimental variables. Such as controlled cellular uptake of fluorophores, photobleaching and clarity of the solution (Hanson et al., 2002). Finally, fluorescence-based BODs are expensive and can only be used once.

On the other hand, electrochemical sensors are highly sensitive, because they are capable of detecting electron flow from one molecule (Nitzan and Ratner, 2003; Shan et al., 2012). This unique sensitivity allows electrochemical systems to overcome many of the problems that optical techniques may encounter, such as optimising dye loading concentration, photobleaching, reactive oxygen species concentration outside the limited range of detection. In addition, electrochemical sensors are faster, more simple to use, and operate at a low cost compared to optical techniques (Wang, 2006). Due to these advantages, electrochemical based systems have gained popularity in many biological applications, such as BODs (Jordan et al., 2013; Saito et al., 2006; Suzuki et al., 2001), fruit fly brain signal studies (Makos et al., 2009) and glucose (Nie et al., 2010), hydrogen peroxide (Chen et al., 2013) and nitric oxide (Griveau and Bedioui, 2013) sensing.

The current electrochemical BOD systems include the use of Clark-type electrodes (Suzuki et al., 2001), microchannel (Saito et al., 2006) and the use of ferricyanide as a mediator in the assay (Jordan et al., 2013; Pasco et al., 2004). The BODs that use the Clark-type electrode system are based on using platinum electrodes pre-coated with a Teflon membrane, allowing oxygen to diffuse through and be measured in real-time. The major setback with this system is that the Teflon membrane limits the amount of oxygen that can diffuse to the electrode, reducing the sensitivity of the sensor. Additionally, formation of platinum oxide means that the system is not fully biocompatible for live cell studies due to its toxicity (Suzuki et al., 2001). The microchannel system is highly sensitive, capable of monitoring single cell metabolism in a real time assay. However, it is relatively expensive and time consuming to fabricate the microchannels (Saito et al., 2006). The BOD assay that makes use of ferricyanide, involves incubating the cells with ferricyanide for one hour in a deoxygenated environment. The bulk concentration of ferricyanide that has been reduced by the cells is then measured after a 1 h period. This is a non-real time assay, which means key cellular events can be missed. For example some molecules are toxic early on but with subsequent passage of time cells can recover. Therefore, these early events can be missed with the standard plat viability assay including presently using BOD (Jordan et al., 2013; Pasco et al., 2004). This is important in circumstances such as drug development as this early toxicity is to be avoided.

The aim of our study was to develop a new electrochemical assay to monitor the biooxygen consumption rates on an unprecedented time-scale. Additionally, a system that is easy to use, with high sensitivity and accuracy, and cost-effective. The primary process to achieving this was to conduct a series of experiments to identify a suitable mediator based on ferrocene derivatives for the specific detection of oxygen. Ferrocene derivatives are commonly used as electrochemical mediators as they have rapid rates of electron transfer, making it ideal for real-time monitoring (Cass et al., 1984). Moreover, ferrocene has an electrochemically reversible one-electron redox behaviour that displays pH-independent redox potentials and non-auto-oxidation of the reduced form (Cass et al., 1985). These unique properties of ferrocene have made it a popular choice as a mediator used in a wide range of electrochemical studies, including in the sensing of glucose (Cass et al., 1984; Schuhmann, 1993, 1995) and development of dehydrogenase-(Serban and El Murr, 2004), oxidases- and other oxidoreductase enzyme-based biosensors (Dicks et al., 1986).

Although ferrocene derivatives have been shown to be capable of interacting with superoxides generated in an aqueous solution, to the best of our knowledge there are no known reports of ferrocene being used to sense aerobic cellular respiration. Thus, we investigated the electrochemical ability of FcA and ferrocene-methanol (FcMeOH) to report on the presence of oxygen. We then perform scan rate studies to investigate the kinetics to establish the optimal mediator to use in cellular studies. The best mediator was then used to report on the oxygen concentration in solution in the presence of cells.

## 2 Materials and methods

### 2.1 Chemicals

LB agar plates, ferrocene carboxylic acid and glucose were purchased from Sigma–Aldrich. LB broth was purchased from Fisher and ferrocene methanol was purchased from Acros Organics. All solutions prepared were oxygenated by being exposed to air in atmospheric conditions. There was no artificial oxidation of the solution performed.

### 2.2 *Escherichia coli* DH5-α culture

*E. coli* DH5-α was stored on LB agar plates at 4 °C. *E. coli* were sub-cultured in liquid LB broth medium the day before the experiment was performed. The LB broth was allowed to grow for 18 hours at 37 °C on a shaker at 200 rpm in baffled flasks. Bacteria were sub-cultured at 1:21 dilution in fresh LB broth in baffled flasks and incubated at 37 °C on a shaker for 2 h. Bacteria were subsequently harvested by centrifuging at 3261×*g* and washed twice in 10 mL of sterile phosphate buffer solution (PBS) (50 mM PBS, pH 7.3 containing 0.1 M KCl) before re-suspending in PBS. Cell density measurements were performed at OD_600_ _nm_ using a Cecil CE1020 UV spectrometer. The cell suspension was kept on ice for up to 5 h until cells were needed for assaying.

### 2.3 Electrochemical measurements

An electrochemical cell consisting of a Ag/AgCl reference electrode, 3 mm diameter glassy carbon working electrode and platinum counter electrode were used in the cyclic voltammetric studies. A Gamry 600 potentiostat with data acquisition software was used for electrochemistry experiments. The glassy carbon electrode was polished with 50 nm alumina powder for 5 min prior to each cyclic voltammogram (CV) being performed. Cyclic voltammetric studies were performed on solutions of 2 mM of ferrocene carboxylic acid (FcA) and ferrocene methanol (FcMeOH) in PBS. The voltammograms were recorded using an initiating potential of 0 V with a switching potential of 0.6 V and an end potential of 0 V. CVs in the absence of *E. coli* were generated at scan rates from 5 to 2000 mV s^−1^ for FcA and FcMeOH in the presence of air (oxygenated). Additionally, cyclic voltammetry was performed on solutions containing FcMeOH and FcA that were purged with oxygen free nitrogen for removal of air (deoxygenated) at scan rates of 5–40 mV s^−1^ and 5 mV s^−1^, respectively.

#### 2.3.1 Electrochemical determination of bacterial cell numbers

Before each assay, 0.5, 1, 1.5, 2, 2.5, 3, 3.5, and 4 OD/mL of *E. coli* were pre-incubated with 10 mM of glucose in PBS at 37 °C on a shaker in conical flasks for 30 minutes. These solutions were then diluted by half. FcA was then added to give a final concentration of 2 mM and cyclic voltammograms (CVs) were recorded at 5 mV s^−1^.

### 2.4 Cytotoxicity measurements

A 5 mL stock solution containing *E. coli* (4 OD_600_ _nm_) was prepared in PBS, containing 10 mM glucose as the growth substrate and HPLC grade ethanol at final concentrations of 0, 2.5, 5, 10 and 12.5%, v/v. This *E. coli* suspension was then incubated in a shaking incubator at 37 °C for 1 h. OD (Optical density) measurements were taken before and after incubation to verify if any cellular growth occurred during the 1 h incubation. For electrochemical interrogation, a 3.5 mL sample of the incubated *E. coli* suspension was added to 3.5 mL of 4 mM FcA in PBS. This gave a final working assay concentration used in cyclic voltammetric studies of *E. coli* at an OD of 2 and FcA at a concentration of 2 mM. Cyclic voltammetry was then performed at a scan rate of 5 mV s^−1^. For agar plate growth assays, the *E. coli* suspension was diluted to 1:1000, 1:100,000, 1:500,000 and 1:1,000,000 prior to seeding onto the agar plates. These agar plates were then incubated for 20 h at 37 °C followed by colony counts after the incubation period.

### 2.5 Fresh water sample testing

Algae rich fresh water samples were collected from two different water sources, namely from a canal and from a stream (Vale water). None living organism controls (acellular controls) were prepared by filtering the water samples through a 0.5 µm filter. 3.5 mL of each sample was mixed with 3.5 mL of 4 mM FcA giving a final concentration of 2 mM FcA. Cyclic voltammetry was then performed at a scan rate of 5 mV s^−1^.

## 3 Results and discussion

### 3.1 Comparison of the ability of FcMeOH and FcA to electrocatalyse the oxidation of reactive oxygen species

**Fig. 1:**
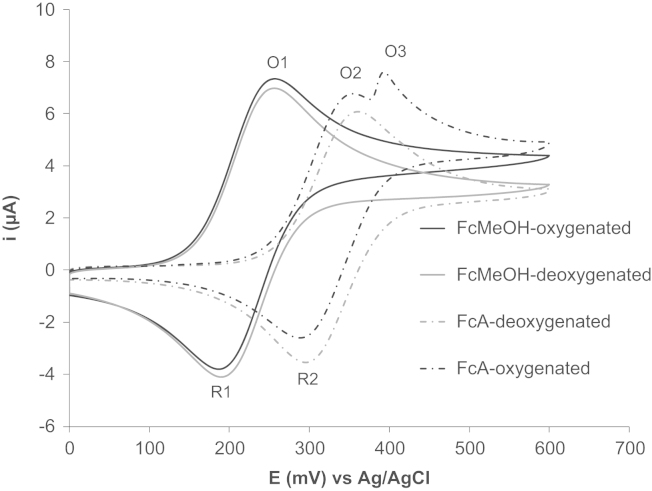
Typical cyclic voltammograms recorded from PBS solutions containing 2 mM FcA or 2 mM FcMeOH in the absence of air (deoxygenated) and the presence of air (oxygenated).

Understanding the properties of an electrochemical mediator is essential in the decision making process for choosing an appropriate mediator for electrochemical-cell studies. Cyclic voltammetry was used to investigate the electrochemical characteristics of FcA and FcMeOH in order to establish the most suitable mediator for probing bacterial aerobic respiration. In preparation for the respiration studies of cells, it was important to ascertain the effect of oxygen on the generated cyclic voltammograms for the mediators FcA and FcMeOH. Therefore, cyclic voltammetry was performed on solutions of PBS containing 2 mM FcA or FcMeOH exposed to oxygen and solutions degassed with oxygen-free nitrogen for the removal of oxygen from the electrolyte. Typical CVs obtained are shown in Fig. 1. Importantly, there is a major difference between the CVs recorded from solutions containing FcA and FcMeOH in the presence of air. When CVs were generated in the presence of oxygen and FcMeOH only one oxidation peak was obtained at a mean peak potential of 257 mV (Fig. 1 O1). However, when CVs were recorded in the presence of FcA and oxygen two oxidation peaks were observed (Fig. 1 O2 and O3) at mean peak potentials of 352 mV and 394 mV. A plot of the magnitude of peak currents obtained from peaks O1 and O2 (Fig. 1 O1 and O2) versus the square root of scan rate yields a proportional relationship indicative of a diffusion controlled process (Fig. S1). This is typical of a simple one electron transfer process in which there is no current contribution from an electrocatalytic process.

**Fig. 2:**
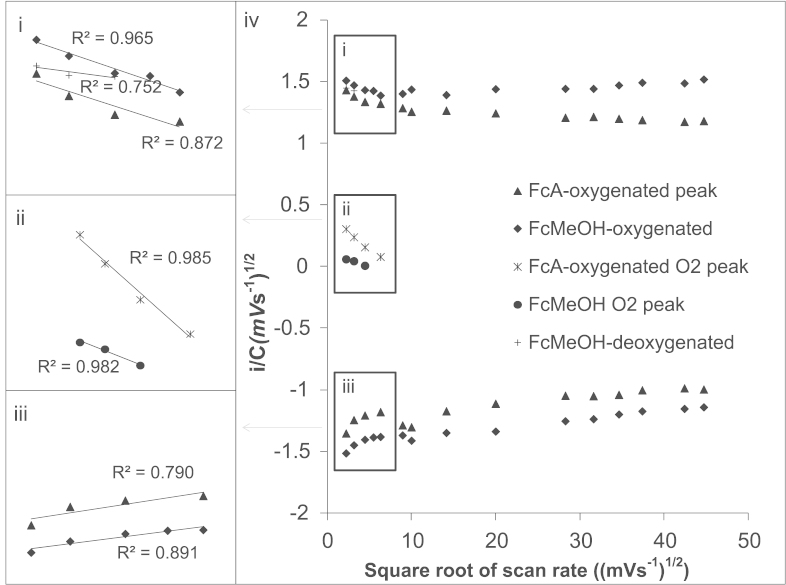
Current functional plot of *i*_pa_ and *i*_pc_ for FcA and FcMeOH; peak currents obtained from cyclic voltammograms recorded from solutions of FcMeOH-oxygenated ◆, FcMeOH-deoxygenated (+), non-electrocatalytic FcA-oxygenated (Fig. 1 O2) ▲, FcA-oxygenated O_2_ electrocatalytic peak ⁎ (Fig. 1 O3), difference between peak currents obtained for FcMeOH-oxygenated and FcMeOH-deoxygenated which termed the electrocatalytic O_2_ peak ● <20 mV s^−1^). Insets represent current values at lower scan rates.

Fig. 1 also indicates that the magnitude of peak current (Fig. 1 O1) for FcMeOH and FcA (Fig. 1 O3) is dependent on the presence of oxygen. This was shown by comparing the oxidation peak current obtained from CVs logged in the presence of FcMeOH in the presence (Fig. 1 O1-oxygenated, mean=6.75 μA, ±1 SD=0.037) and absence (Fig. 1 O1-deoxygenated, mean=6.48 μA, ±1 SD=0.053) of oxygen. This comparison showed that CVs recorded in the presence of oxygen resulted in an increase in the magnitude of the O1 peak of approximately 200 nA and an equivalent decrease in the reduction peak occurred (Fig. 1). The magnitude of the oxidation peak current measured from CVs recorded with solutions of FcMeOH which can be attributed to oxygen, is equal to the difference between the peak current obtained in the presence and absence of oxygen. We conclude that any current generated which is associated with the presence of O_2_ is convoluted with the normal FcMeOH electrochemistry. Additionally, results from the current function plots for FcMeOH (Fig. 2) also supports this proposition as the correlation coefficients obtained at low scan rates prior to subtracting the deoxygenated peak (*R*^2^=0.965) lies between the deoxygenated correlation coefficient value of *R*^2^=0.752 and O_2_ electrocatalytic current correlation coefficient value of *R*^2^=0.982. However, when cyclic voltammetry was performed with solutions of FcA in a deoxygenated solution, the O3 peak current associated with the presence of oxygen (Fig. 1 O3) was no longer observed. We conclude that the O3 peak (Fig. 1) observed in the CV recorded in the presence of FcA arises from the existence of oxygen in the assay solution. For simplicity the current that is attributed to the presence of oxygen is termed the O_2_ electrocatalytic current.

The proposed mechanism which gives rise to the observed O_2_ electrocatalytic current generated in the CVs recorded in the presence of FcA and FcMeOH is attributed to the oxygen which is electrochemically reduced at 0 V forming a superoxide anion (O_2_^−^) and is supported by Cassidy (Cassidy et al., 1999). This conclusion is elucidated by the fact that on removal of the oxygen there is a decrease in peak current observed in CVs logged with solutions of FcMeOH (Fig. 1) and removal of the electrocatalytic O_2_ peak in CVs documented for FcA. The O_2_^−^ subsequently chemically oxidises water forming the reactive oxygen species hydroxyperoxyl (HO_2_^−^) and a hydroxyl ion (OH^−^). In the positive scan Fc is electrochemically oxidised to Fc^+^. We suggest the OH^−^ and HO_2_^−^ generated reduces the Fc^+^ to Fc and this is subsequently electrochemically reoxidised. This leads to a catalytic enhancement in the magnitude of the oxidation peak current as the concentration of reduced Fc is increased in the presence of oxygen. Moreover, it is well understood that peak current is proportional to concentration of redox molecules under investigation and explains why in deoxygenated solutions a decrease in the observed electrocatalytic peak oxidation current obtained from CVs reported for FcMeOH and FcA solutions. The mechanism we propose is an electrochemical–chemical–electrochemical (E–C–E) system. It can be presumed that the chemical steps must be relatively slow because with increasing scan rate there is no increase in the observed O_2_ peak electrocatalytic current obtained for CVs generated in the presence of FcMeOH above 20 mV s^−1^, and 40 mV s^−1^ for FcA. We suggest that the reason for this difference is because the rate at which the chemical step occurs is slower with the FcMeOH than the FcA. This is also supported by Fig. S2 in which we show the actual charge transfer coefficient for the electrocatalytic oxidation is fast for the FcA when compared to FcMeOH. Consequently, FcA produces a larger electrocatalytic current under the same condition.

We suggest that the difference in behaviour observed for FcA and FcMeOH in terms of the position of peak potentials is caused by the different functional groups. FcA contains a carbonyl and FcMeOH a hydroxyl. The electron withdrawing effect of the carbonyl group adjacent to the cyclopentadienyl ring leads to a lowering of the electron density around the iron (Fe^2+^) centre. This means the ferrocene requires a larger over-potential to be oxidized and evidence for this behaviour has been reported by others for different ferrocene derivatives (Hocek et al., 2004; Wu et al., 2006). This would decrease the ability of the Fe^2+^ to lose electrons, therefore, as observed, a higher potential is required to electrochemically oxidize FcA than FcMeOH, resulting in FcMeOH having a lower redox potential than FcA **(**Fig. 1**)** (Hocek et al., 2004; Wu et al., 2006). This behaviour also explains why the O_2_ electrocatalytic peak for FcA is de-convoluted, whilst for FcMeOH it is convoluted, and occurs at higher potentials than the simple non-electrocatalytic current for the FcA. This difference is caused by the fact that after the Fc is oxidized into Fc^+^, the Fe^3+^ centre interacts with the surrounding OH^−^ and HO_2_^−^. We propose that the Fe^3+^ center is then instantaneously reduced by the reactive oxygen species forming an adduct and we hypothesize that the carbonyl group on the FcA makes the adduct relatively stable. This causes a further lowering of the electron density on the iron centre when compared to FcA alone. As a result, an even higher oxidation peak potential is needed to oxidise FcA resulting in the separation of the O_2_ electrocatalytic peak. On the other hand, the FcMeOH adduct is not stabilized and instantaneously oxidises. Therefore, the catalytic signal seen for FcMeOH is not separate from the normal FcMeOH signal when compared with the FcA.

In order to understand the factors controlling the mechanism and to provide further evidence that the electrocatalytic behaviour was occurring via the mechanism proposed, investigations into the kinetics of the reaction were investigated by performing cyclic voltammetry with solutions containing FcA and FcMeOH at varying scan rates in both oxygenated and deoxygenated solutions. Peak currents obtained from cyclic voltammograms generated at varying scan rates were then plotted into a current functional plot (Fig. 2). At higher scan rates there is no relationship with current function (Fig. 2iv). This lack of relationship at relatively fast scan rates is indicative of a diffusion limited process and supports the data obtained in Fig. S1. However, when we analyze the plot at slower scan rates there is a deviation from this behaviour when oxygen is present both for FcA and FcMeOH (Fig. 2i–iii) and is indicative of an electrocatalytic process (Nicholson and Shain, 1965).

In the absence of oxygen, the current function plot for FcMeOH (<20 mV s^−1^) yields a correlation coefficient value of *R*^2^=0.752 (Fig. 2i), suggesting the current is diffusion limited. On the other hand, in the presence of oxygen the electrocatalytic current obtained for FcMeOH (obtained by subtracting the peak current obtained in the presence of oxygen minus the peak current obtained in the absence of oxygen) is directly correlated to scan rate in the current function plot with a correlation coefficient value of *R*^2^=0.982, indicative of a non-diffusion limited process (Fig. 2ii). This relative large correlation provides supporting evidence that the electrocatalytic behaviour is occurring via the proposed E–C–E mechanism and we attribute this deviation to a slow chemical step which is rate limiting.

The correlation coefficient values obtained from the current function plot for the non-electrocatalytic FcA peak (Fig. 1 O2) in the presence of oxygen yields a relatively low correlation coefficient value of *R*^2^=0.872 at low scan rates (Fig. 2i). This indicates that the peak current even at slower scan rates (<40 mV s^−1^) is under diffusion control as expected for a simple 1 electron transfer redox process. Moreover, the current function values obtained for the electrocatalytic O_2_ peak for FcA (Fig. 2ii) yield a correlation coefficient of *R*^2^=0.985 indicating the current is under non-diffusion control and we suggest this arises this peak represents the electrocatalytic oxidation. This deviation from diffusion controlled behaviour is attributed to the chemical step being rate limiting which is similar behaviour to that observed with FcMeOH. Consequently, oxidation peak O2 (Fig. 1) is non-catalytic current arising from the redox events of the FcA alone whereas the electrocatalytic O_2_ peak (Fig. 1 O3) represents the electrocatalytic oxidation of OH^−^ and/or HO_2_^−^.

Combining the data discussed for FcA and FcMeOH, FcA was specifically chosen for the next part of our study for the following reasons: Even though FcMeOH has the lowest *E*_pa_ making it the most favourable mediator in a biological system, the slower chemical electron transfer kinetics in the reduction of FcMeOH in the presence of oxygen would make the system less sensitive. The unique and separate electrocatalytic O_2_ peak obtained for FcA would simplify the system of study because there is no need to conduct two cyclic voltammetry studies in the presence of and absence of oxygen in order to obtain the electrocatalytic signal.

### 3.2 Using FcA-mediated system to detect *E. coli* concentration

**Fig. 3:**
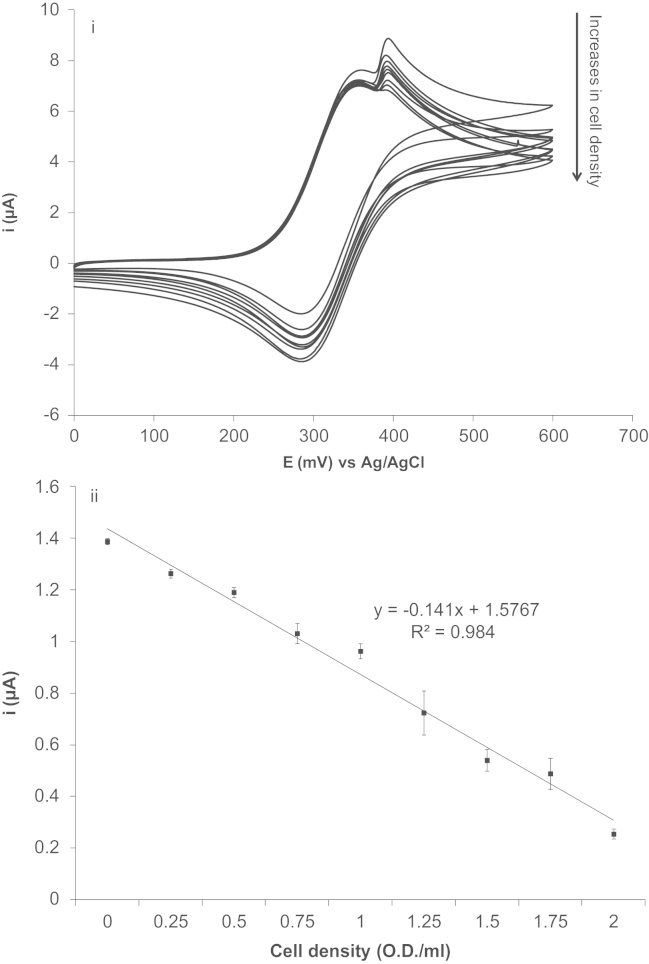
(i) Typical CVs obtained for the first of the two cycles for solutions containing different *E. coli* concentration of 0, 0.25, 0.5, 0.75, 1, 1.25, 1.5, 1.75, and 2 OD/mL with 2 mM FcA. (ii) A plot of OD_600_ _mn_ versus electrocatalytic O_2_ peak current obtained from cyclic voltammograms in (i). All CVs were performed at a scan rate of 5 mV s^−1^ (*n*=8, ±1 SE).

*E. coli* (strain DH5α) was used to characterise the ability of FcA to monitor cellular respiration via oxygen concentration. Optical density measurements were calibrated by determining the number of *E. coli* cells, using a haemocytometer, in a solution that had a 1 OD_600_ _nm_/mL. This was followed by serial dilution of the stock *E. coli* solution to the appropriate concentrations in PBS. The cell solution was subsequently pre-incubated with 10 mM of glucose then mixed with a final concentration of 2 mM FcA. Two consecutive cyclic voltammograms were performed on solutions containing varying numbers of cells (0, 0.25, 0.5, 1.75, 1, 1.25, 1.5, 1.75 and 2 OD/mL) and Fig. 3 summarises the first of the two consecutive cyclic voltammograms conducted to demonstrate the magnitude of the FcA electrocatalytic O_2_ peak. As expected, an increase in number of *E. coli* leads to an increase in oxygen consumption, and therefore, a decrease in the electrocatalytic O_2_ peak current. In addition, the decrease in the electrocatalytic O_2_ peak current is directly proportional to the increase in *E. coli* cell numbers.

**Fig. 4:**
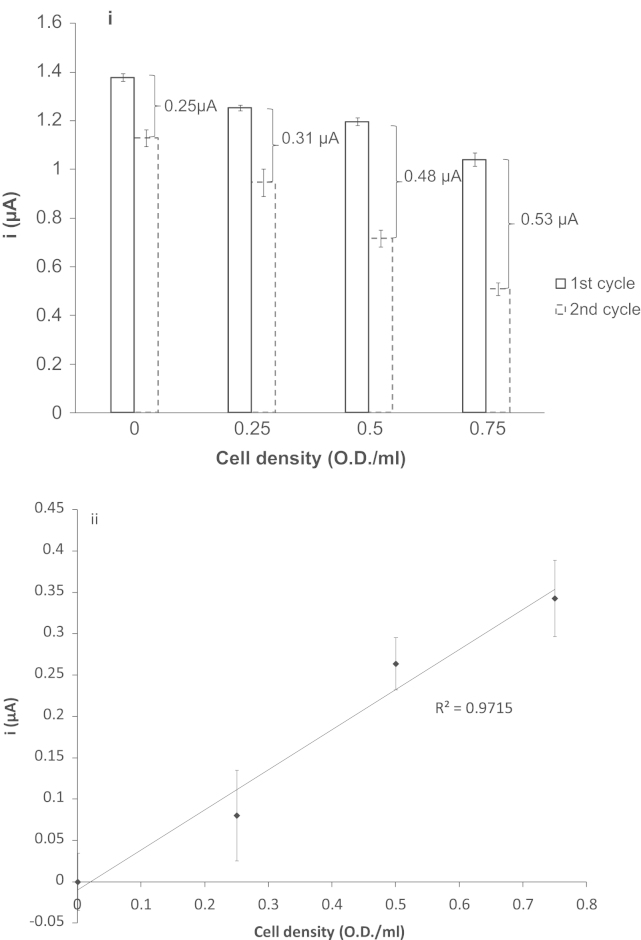
(i) A histogram of the magnitude of the mean electrocatalytic O_2_ peak current obtained from the cyclic voltammogram on the first cycle and second cycle (0, 0.25, 0.5, and 0.75 OD/mL). (ii) The first and second electrocatalytic O_2_ peak current differences minus 18% (negative control − cyclic voltammetry studies conducted in the absence of *E. coli*), (*n*=5, ±1 SE). In addition to the detection of oxygen consumption, we have shown our system to be compatible in the measurement of oxygen increase in solution (see S1.2).

During the assay the *E. coli* would be continuously consuming oxygen, and this could be monitored in near real-time by measuring the change in the catalytic peak current between two consecutive cycles. The electrocatalytic O_2_ peak current generated on the first cycle and the second cycle is exactly one cyclic voltammetric scan apart at a fixed scan rate of 5 mV s^−1^. A plot of the electrocatalytic O_2_ peak current documented from the two cycles at the varying cell concentrations can be observed in Fig. 4i. Assay solutions containing higher cell numbers resulted in a larger difference in the magnitude of the current between the cycles. It is also worth mentioning that there is always an 18% drop in the magnitude of the electrocatalytic O_2_ peak observed between the first and second cyclic voltammteric cycle in the control study (cyclic voltammetry conducted in the absence of *E. coli*). This indicates that oxygen from the atmosphere cannot dissolve into solution at sufficient rates to replenish electrochemically consumed oxygen. It will therefore not interfere with measurement of O_2_ in cellular assays. Therefore, any current decrease measured that was greater than 18% between the first and second electrocatalytic O_2_ peak can be assigned to oxygen consumption by the *E. coli*. Moreover, a decrease in the peak current obtained for the electrocatalytic O_2_ peak between the two cyclic voltammetric cycles is directly proportional to the increase in *E. coli* cell numbers (Fig. 4ii). Using Eq. (1), the oxygen consumption rate down to a single cell level (cells per second) was calculated to be approximately 1.12×10^−17^ mol cell^−1^s^–1^, which is similar to the values published in the literature (4.31×10^−20^ mol cell^−1^s^–1^) for *E. coli* strain *K21* (Saito et al., 2006). The difference in oxygen consumption rate observed in our study using *E. coli* strain *DH5-*α and the *K21* strain is likely due to the difference in metabolic demands between the two *E. coli* strains and the different culture conditions resulting in the cells being at different stages of the growth cycle. More importantly, in this study, we demonstrated for the first time the simplicity and the accuracy and sensitivity of the FcA-mediated system:

(1) $$\begin{array}{l} (i)\;O_{2}\;\mathrm{consumption}\;(\mathrm{mol}\;{\mathrm{cell}}^{-1}\;s^{-1})=\frac{(\mathrm{moles}\;\mathrm{of}\;O_{2}\;\mathrm{from}\;\mathrm{1st}\;\mathrm{cycle}\;\times X)-\mathrm{moles}\;\mathrm{of}\;O_{2}\;\mathrm{from}\;\mathrm{2nd}\;\mathrm{cycle}}{\mathrm{time}\;(s)\;\times \mathrm{number}\;\mathrm{of}\;\mathrm{cells}}\\ (\mathrm{ii})\;\mathrm{moles}\;\mathrm{of}\;O_{2}\;\mathrm{from}\;\mathrm{each}\;\text{cycle}\;=\left(\frac{i_{p}}{(2.99x10^{5})n{\left(\propto n_{a}\right)}^{1/2}AD^{1/2}v^{1/2}}\right)\times \mathrm{volume}\;\;\text{in}\;{\mathrm{cm}}^{3} \end{array}$$

where *X*=percentage of current drop between 2 cycles. This percentage drop varies with different electrolytes, and is obtained by doing a non-living organism control e.g. filtering the samples; *i*_*p*_=the peak current (A); *n*=the number of electrons; *α*=the transfer coefficient (see S1.1); *n*_*a*_=the number of electrons in the rate limiting step; *A*=the surface area of the electrode (cm^2^); *D*=the diffusion coefficient (cm^2^ _S_^−1^) calculated from the non-electrocatalytic FcA peak (Fig. 1 O1) using the Randles–Sevcik equation for reversible system (see S1.1); *ν*=the scan rate (V s^−1^).

### 3.3 Electrochemical cytotoxicity assay

To confirm that the FcA-mediated assay can accurately report on the metabolic rate of cells and to demonstrate the wide capabilities of the developed assay the FcA was used to detect the toxicity of a model toxin ethanol. Cyclic voltammetric studies were conducted using solutions containing a fixed number of *E. coli* (4 OD/mL) and incubated with different concentrations of ethanol (0, 2.5, 5, 10, and 12.5%, v/v) and 2 mM FcA. Ethanol was used because it is cytotoxic to *E. coli* through the disruption of plasma membrane (Dombek and Ingram, 1984). We hypothesized that an increase in the ethanol concentration would lead to a decrease in the number of viable cells. Consequently, this leads to a decrease in oxygen consumption and therefore an increase in the generated electrocatalytic O_2_ peak current. In addition, a control was performed in which the optical density of *E. coli* was measured post-incubation with ethanol to ensure that the same number of cells were still present prior to performing cyclic voltammetric studies to confirm that any difference was not due to bulk changes in cells concentration. Moreover, our electrochemical assay results for analyzing the toxic effects that ethanol had on the cells were compared to a standard viable agar plate method of toxicity testing to enable validation of our system.

**Fig. 5:**
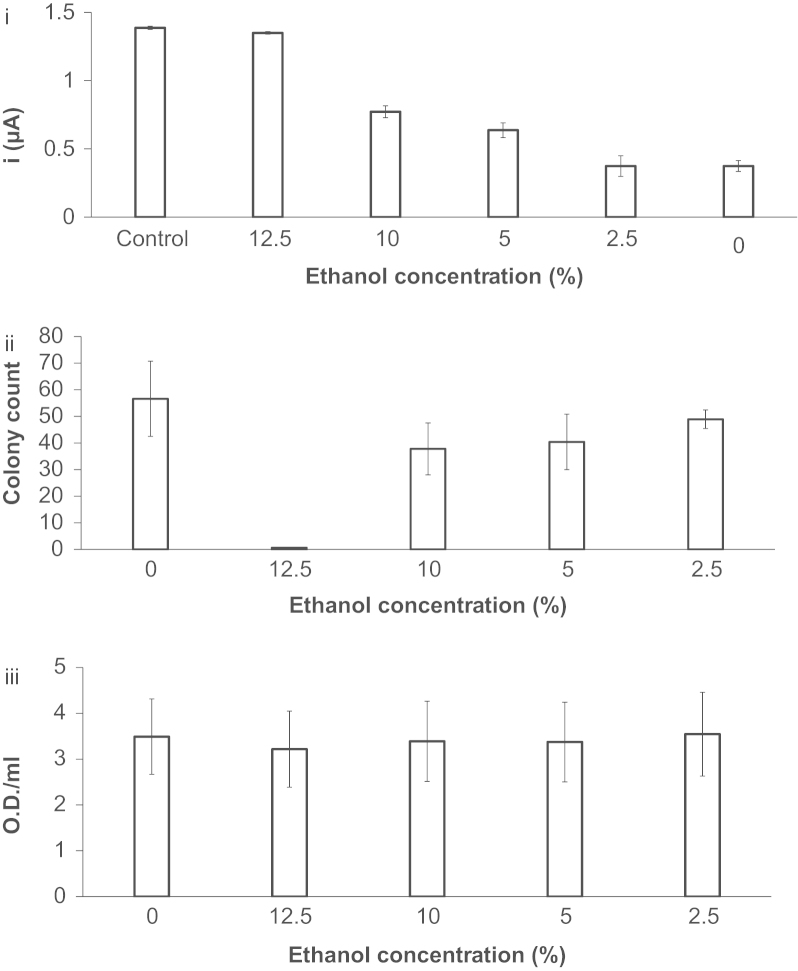
(i) The magnitude of the mean electrocatalytic O_2_ peak current difference obtained between the first and second cyclic voltammograms generated from solutions containing cells at 4 OD/mL at varying concentrations of ethanol (0, 2.5, 5, 10, or 12.5%, v/v) and 2 mM FcA. A cyclic voltammogram was recorded in the absence of *E. coli* which acted as a negative control (*n*=5, ±1 SE). (ii) The mean colony counts post-ethanol incubation of same batch of cells used in the cyclic voltammetry study (*n*=14–16, ±1 SE). (iii) The mean optical density measured by UV spectrophotometer at 600 nm post-ethanol incubation of the same batch of cells used for cyclic voltammetry studies and agar plate bacterial growth assay (*n*=5, ±1 SE).

The electrocatalytic O_2_ peak currents generated in CVs obtained in the presence of ethanol and cells are summarized in Fig. 5i. In the presence of relatively high ethanol concentrations a decrease in oxygen consumption and consequently increase in the electrocatalytic O_2_ peak current was observed. As expected the magnitude of the peak current was inversely proportional to the increase in ethanol content. A complete loss in oxygen consumption was determined at 12% (v/v) ethanol, indicating that no viable cells since the electrocatalytic O_2_ peak current of the negative control (cyclic voltammetry studies in the absence of cells) is approximately the same as cell incubated with 12% (v/v) ethanol. In addition, the number of viable cells was confirmed by the growth assay (Fig. 5ii) with the increase in ethanol concentration, there was a decrease in viable cell numbers and a complete loss in cell viability at 12% (v/v) ethanol concentration matching the electrochemistry results. Moreover, the optical density study confirms the number of cells pre- and post-ethanol incubation was consistent, see Fig. 5iii. This further demonstrated the biological compatibility and sensitivity of the FcA-mediated system. Moreover, a key advantage of the toxicity assay developed was that it is much more rapid compared to the standard plate viability assay which takes 24 h to perform compared to seconds with the our electrochemical method. In addition, our system reports sub-lethal toxicity which is missed by the plate viability assay as we see that there is no significant difference at concentrations of ethanol obtained equal to and below 10%. This is because over the 24 h period the cells can recover from the lower concentration and therefore the sub-lethal toxicity is missed which is reported by the electrochemistry assay. This is important and demonstrates a key advantage of our toxicity assay as pharmaceutical companies are interested in avoiding sub-lethal toxicity. The developed electrochemical method (Fig. 5i) is also more accurate than the plate viability assay as indicated by the much larger standard error bars (Fig. 5ii).

**Fig. 6:**
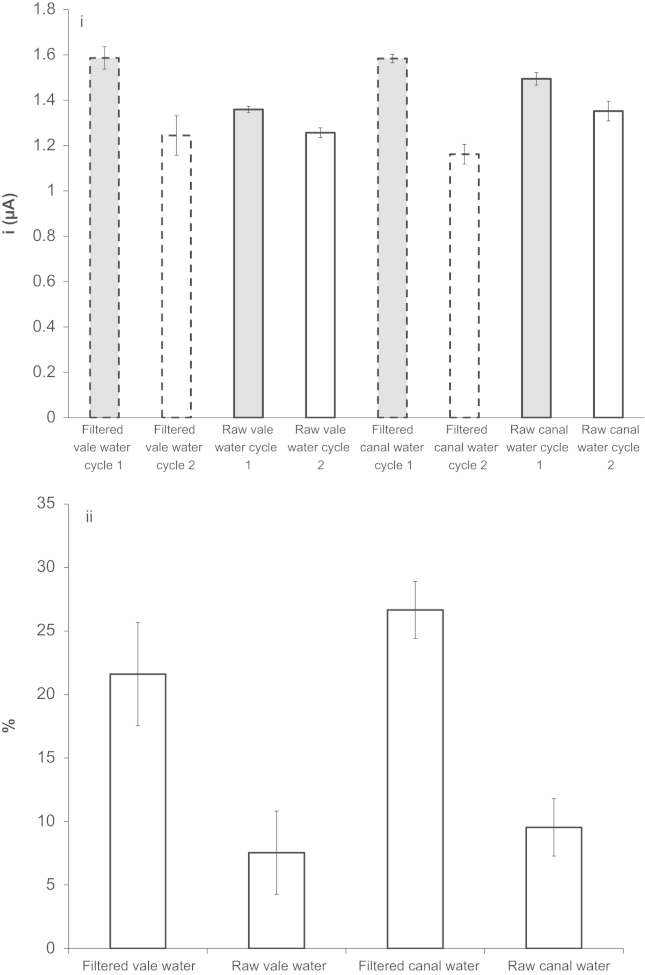
(i) A histogram of the magnitude of the mean electrocatalytic O_2_ peak current obtained from recorded cyclic voltammograms of the first and second cycle (filtered generated from solutions of raw Vale/canal water samples (*n*=3)). (ii) A histogram of the mean percentage electrocatalytic O_2_ peak current drop between first cycle and second cycle of the logged cyclic voltammograms (water control (*n*=2), filtered and raw Vale/canal water samples (*n*=3)) (±1 SD).

In order to investigate further the applicability of the developed FcA mediated oxygen demand assay for multiple applications, experiments were performed with Algae rich fresh water samples. These were collected from nearby natural water resources, namely from a canal and from a stream (Vale water). These water sources were filtered to remove all living organisms and decaying matter, serving as acellular controls. This allowed us to obtain the standard percentage current drop due to the limitation of oxygen diffusion in different electrolyte environments. From simple observation, the water sample collected from the canal appeared as a darker shade of green than the water sample from the Vale, suggesting that the former had higher algae content. After filtration, both water samples became clear. CVs were generated for acellular controls and raw water samples from the canal and Vale stream and the O_2_ electrocatalytic peak currents were measured. A summary of the current change and percentage current change between the two cyclic voltammetric cycles for both raw samples and control samples are plotted in Fig. 6. Using Eq. (1) part ii, the electrocatalytic O_2_ peak current of the first cycle can be used to calculate the overall oxygen contained in the sample. For the Vale and canal water samples a total oxygen concentration of 341.6 µM and 351.3 µM was calculated, respectively. Interestingly, during the time frame of the 2 consecutive cyclic voltammteric scans performed on the environmental water samples, the oxygen content increased which was opposite to the result observed for *E. coli*. In the case of *E. coli* samples, the cells were consuming oxygen in the solution therefore the decrease of the electrocatalytic O_2_ peak at the second cycle is greater when comparing to the acellular controls. That is if one calculates the percentage change in current between the first cycle from the second cycle of acellular control, and then subtract the equivalent percentage change calculated from the *E. coli* working sample at OD 0.75, a relative negative change when compared to the acellular control of approximately of −33% is calculated (18–51%=−33% values obtained from Fig. 3ii). This value of −33% represents oxygen consumption. For the case of the raw water samples obtained from the Vale and canal an approximate relative change, when working samples were compared to acellular controls, of +14% and +18% were calculated, respectively. The larger value obtained for the canal sample of 18% is indicative a high algae content as previously observed by a deeper green colour of the sample. Algae are capable of photosynthesizing and producing oxygen and consequently this experimentally proves that the canal water had a higher algae content than the Vale water. In this study, the electrocatalytic O_2_ peak obtained from the second CV generated in the presence of the raw algae rich water samples showed a relatively much smaller decrease in the current when comparing it to the acellular controls. This indicates that there is an increase in oxygen content over time for the raw water samples. By inputting the data into Eq. (2), where *X* is defined as in Eq. (1), an increase in oxygen level of 2.522×10^−9^ mol s^−1^ and 3.422×10^−9^ mol s^−1^ was obtained from the vale and canal water, respectively. These experiments demonstrated that FcA mediated oxygen demand assay can also be used for detecting increases in oxygen content in solution over time, not just designed for detecting oxygen consumption:

(2) $$O_{2}\;\mathrm{increase}\;(\mathrm{mol}\;s^{-1})=\frac{(\mathrm{moles}\;\mathrm{of}\;O_{2}\;\;\text{from}\;\;\mathrm{1st}\;\mathrm{cycle}\times X)-\mathrm{moles}\;\mathrm{of}\;O_{2}\;\mathrm{from}\;\mathrm{2nd}\;\mathrm{cycle}}{\mathrm{time}\;(s)\;}$$

## 4 Conclusions

In summary, we have unveiled here the unique behaviour of FcA to electrocatalytically report on oxygen concentration via its interaction with OH^−^ and HO_2_^−^ and provide a detailed mechanistic insight into the reaction. The ability of FcA to monitor oxygen was taken advantage of to develop a rapid cellular respiration assay which could be monitored in near real-time which is faster than any comparable BOD assay. This assay was shown to be able to report accurately on the cell numbers present and was adapted to be used as a rapid toxicity assay. Additionally, we showed that the oxygen demand assay developed can be used in a complex water environment to determine total oxygen concentration and show that the method is sensitive to oxygen increases. It is envisaged (Rawson et al., 2013) that the developed assay has potential to impact on fields and industries ranging from environmental toxicology through to pharmaceutical and agrochemical industries as demonstrated by the shortening of a commercially available bioxygen demand assay down to minutes rather than 5 days.
