# Supplementary material for: Real-time electrocatalytic sensing of cellular respiration
Source: Biosens Bioelectron. 2014 Jul 15;57(100):303–9. doi: 10.1016/j.bios.2014.01.059 (PMC3990025; doi:10.1016/j.bios.2014.01.059)
Supplement: Supplementary file 1 — Supplementary data [file mmc1.doc]

**Real-Time Electrocatalytic Sensing of Cellular Respiration**

**Nga-Chi Yip^1^, Frankie J Rawson^2^, Chi Wai Tsang^3^, Paula M Mendes^1^***

**Supporting Information**

^1^School of Chemical Engineering,

University of Birmingham,

Edgbaston, Birmingham B15 2TT.

^2^Laboratory of Biophysics and Surface Analysis

School of Pharmacy

University of Nottingham

University Park

Nottingham, NG7 2RD

^3^School of Chemistry

University of Birmingham,

Edgbaston, Birmingham B15 2TT.

Professor Paula M Mendes

School of Chemical Engineering

University of Birmingham

Edgbaston, Birmingham, B15 2TT (UK)

Tel: Int. code +(121) 414-5343

Email: p.m.mendes@bham.ac.uk

# Correspondence Address

**S1.1 Electrochemical characterisation of FcA and FcMeOH**

All cyclic voltammetric studies described were conducted in PBS with a final concentration of 2 mM FcA or 2 mM FcMeOH. Typical CVs obtained for FcA and FcMeOH conducted on the open bench (oxygenated) at a scan rate (SR) of 5 mVs^-1^ is shown in **Figure 1.** The mean separation in peak potential (∆E_p_) for FcA and FcMeOH is 66 mV (±1SD=1.572) and 70 mV (±1SD=4.561), respectively. The values for the mean oxidation peak current over reduction peak current (i_pa_/i_pc_) are 0.993 (±1SD=0.022) and 0.997 (±1SD=0.007), respectively. These values indicate that both FcA and FcMeOH are displaying a quasi-reversible electrochemical behavior ([Laschi et al. 2009](#_ENREF_3); [Nie et al. 2010](#_ENREF_5)). The mean peak potential (Ep) values for FcMeOH are 257 mV (±1SD=3.194) for the oxidation peak and 187 mV (±1SD=1.518) for the reduction peak, whereas in the case of FcA, they are 353 mV (±1SD=0.643) for the oxidation peak and 287 mV (±1SD=1.692) for the reduction peak. These values are similar to the values reported in the literature([Laschi et al. 2009](#_ENREF_3)). Additionally, a second oxidation peak is observed at approximately 394 mV (±1SD=1.510), which is slightly higher than 317 mV reported in Cassidy’s study([Cassidy et al. 1999](#_ENREF_1)) and is likely due to the use of different electrolytes. This study showed FcMeOH requires much less energy to trigger the electrochemical redox event than FcA (See **Figure S2**) as highlighted by the lower peak potential values. This would be advantageous to an electrochemical bioxygen demand assay, since the applied potential for the cyclic voltammteric scans would be lower, and therefore, lowering the risk of influencing cellular membrane potential ([Haberl et al. 2013](#_ENREF_2)), thereby avoiding perturbations of cells metabolism induced by electronic fields. Additionally, it minimises the possibility of encountering problems with interfering species.

A scan rate study was performed with solutions of FcMeOH and FcA and typical peak current obtained at the varying scan rates are plotted in **Figure S1**.The peak currents for both FcA (▲) and FcMeOH (◆) are proportional to the square root of the scan rate. This well-known behavior indicates that the peak current is under diffusion control. We also noted that at higher scan rates the electrochemical O_2_ peak (**Figure 1 peak** O3) observed for FcA is no longer present. We were interested in ascertaining the rate of diffusion of the mediators as this can influence the sensitivity of the system. Using the Randles-Sevcik equation (**i_p_=(2.69x10^5^)n^3/2^AD^1/2^Cν^1/2^**), the diffusion coefficient (D) of FcMeOH and FcA were calculated from 5 mVs^-1^ oxidation peak data (Peak O1 and O3 from **Figure 1 i**). The diffusion coefficient for FcMeOH (D=7.87 x 10^-7^ cm^2^s^-1^) is similar to that reported in the literature (D=2.50 x 10^-7^ cm^2^s^-1^) ([Lovelock et al. 2011](#_ENREF_4)). Whereas, the diffusion coefficient for FcA (D=7.08 x 10^-7^ cm^2^s^-1^) is a magnitude smaller than reported in the literature (D=4.30 × 10^-6^ cm^2^s^-1^) ([Nie et al. 2010](#_ENREF_5)). It is not a surprise that the diffusion coefficient for FcMeOH and FcA obtained in our study are not identical to those reported in the literature, since the viscosity of the room temperature ionic liquids used would greatly influence the diffusion coefficient of the mediator([Lovelock et al. 2011](#_ENREF_4)). We show that FcMeOH has a faster diffusion coefficient than FcA in our study, which could be explained by FcMeOH (Mw=216.06 g mol^-1^) being a smaller molecule than FcA (Mw=274.05 g mol^-1^).

**Figure S1.** Plot of oxidation and reduction peak currents versus square root of scan rate from CVs obtained for FcA and FcMeOH at varying scan rates.

The oxidation peak potential of FcMeOH (**Figure 1** O1 ) and FcA (**Figure 1** O2 and O3) obtained from cyclic voltammograms performed in the presence of oxygen were plotted against logarithm of scan rate (**Figure S2**). As seen from the figure, there were no changes in oxidation peak potential for scan rates up to about 100 mVs^-1^ in the case of FcMeOH (**Figure S2** FcMeOH O1 peak) and FcA non-electrocatalytic peak (**Figure S2** FcA O2 peak). For scan rates beyond 100 mVs^-1^, both peak potentials mentioned changed linearly with log of scan rate with correlation coefficient values of R^2^=0.937 and R^2^=0.955 for FcA-oxygenated non-catalytic peak (**Figure S2** FcA O2 peak) and FcMeOH-oxygenated (**Figure S2** FcMeOH O1 peak). On the other hand, the FcA electrocatalytic peak (**Figure S2** FcA O3 peak) yielded a correlation coefficient value of R^2^=0.964 from very low scan rates (5mVs^-1^ to 40 mVs^-1^). These observations indicated FcMeOH (**Figure S2** FcMeOH O1 peak) and FcA-non-electrocatalytic peak (**Figure S2** FcA O2 peak) were quasi-reversible over the scan rate range of 5 to 100 mVs^-1^ and irreversible beyond 100 mVs^-1^. Whereas in the case of the FcA electrocatalytic process (**Figure S2** FcA O3 peak) is irreversible from 5-40 mV s^-1^.

By comparing the graphical lines for the irreversible behavior we can ascertain that the electrocatalytic current for FcMeOH is convoluted with the non-electrocatalytic current. We can calculate the charge transfer coefficients (αn_a_) for the various peaks by using the following equation in which, ∆Epa/∆log*v* is equivalent to the gradient of the plots obtained in **Figure S2**.

$\frac{\Delta Epa}{\Delta logv}=\frac{2.3RT}{2\alpha naF}$ **Equation S1**

In which *Epa* (V) is the is oxidation peak potential, *v* is scan rate in mV s^-1^, n_a_ is the number of electrons in the rate determining step and α is the charge transfer coefficient in which with increasing faster charge transfer process. R is the standard gas constant, T is the standard temperature in Kelvin at 25^o^C.

The charge transfer coefficient value of 0.28 was obtained for the FcA non-electrocatalytic peak. In contrast, the FcA electrocatalytic peak (**Figure S2** FcA O3 peak) has an α value of 0.77 for one electron. This indicates two electrons are involved in this oxidation. However, due to the nature of FcMeOH electrocatalytic peak and non- electrocatalytic peak being convoluted, the FcMeOH electrocatalytic peak potential just simply cannot be extract for the α value calculation.

**Figure S2**. Oxidation peak potential versus logarithm of scan rate measured for FcMeOH-oxygenated, FcA-oxygenated.

Cassidy, J., O'Gorman, J., Ronane, M., Howard, E., 1999. Note on the voltammetry of ferrocene carboxylate in aqueous solution. Electrochem Commun 1(2), 69-71.

Haberl, S., Miklavcic, D., Sersa, G., Frey, W., Rubinsky, B., 2013. Cell membrane electroporation-Part 2: the applications. Electrical Insulation Magazine, IEEE 29(1), 29-37.

Laschi, S., Palchetti, I., Marrazza, G., Mascini, M., 2009. Enzyme-amplified electrochemical hybridization assay based on PNA, LNA and DNA probe-modified micro-magnetic beads. Bioelectrochemistry 76(1), 214-220.

Lovelock, K.R., Ejigu, A., Loh, S.F., Men, S., Licence, P., Walsh, D.A., 2011. On the diffusion of ferrocenemethanol in room-temperature ionic liquids: an electrochemical study. Physical chemistry chemical physics : PCCP 13(21), 10155-10164.

Nie, Z., Nijhuis, C.A., Gong, J., Chen, X., Kumachev, A., Martinez, A.W., Narovlyansky, M., Whitesides, G.M., 2010. Electrochemical sensing in paper-based microfluidic devices. Lab on a Chip 10(4), 477-483.
